# Effects of dehydroabietic acid on nontarget lipidomics and proteomics of HepG2

**DOI:** 10.3389/fphar.2022.1015240

**Published:** 2022-12-01

**Authors:** Zhikai Xiahou, Jun Han

**Affiliations:** ^1^ China Institute of Sport and Health Science, Beijing Sport University, Beijing, China; ^2^ Beijing Tcmages Pharmaceutical Co.Ltd., Beijing, China; ^3^ Beijing Kangrentang Pharmaceutical Co.,Ltd., Beijing, China

**Keywords:** dehydroabietic acid, liver cancer, proteomics, lipidomics, ACSL3

## Abstract

**Objective:** Studies of the effects of dehydroabietic acid on the multiomics of HepG2 hepatoma carcinoma cells are currently lacking. In this study, the molecular mechanism of the influence of dehydroabietic acid on HepG2 cells was disclosed by studying lipidomics and proteomics. Correlations among multiomics conjoint analysis results were verified.

**Methods:** First, proteomics analysis of HepG2 cells was carried out using dehydroabietic acid. Differentially expressed proteins were screened and analyzed. Pathway enrichment analyses of differential proteins were compared, and the molecular mechanism was disclosed. Second, lipidomics analysis of HepG2 cells was conducted using dehydroabietic acid. The influence of dehydroabietic acid on HepG2 cells was determined on the lipid molecular level. Finally, a conjoint analysis of data related to differentially expressed proteins of ferroptosis and differentially changing lipid molecules was implemented.

**Results:** A total of 260 upregulated and 961 downregulated proteins were screened in the proteomics analysis. The top five significantly enriched pathways included ferroptosis, oxidative phosphorylation, and protein processing in the endoplasmic reticulum. In the lipidomics analysis, 30 significantly differential metabolites with upregulated and downregulated expression were identified, and differentially expressed lipids were mainly related to the metabolism of glyceryl phosphatide. According to the comprehensive multiomics analysis results, real-time quantitative PCR and the enzyme-linked immunosorbent assay (ELISA), ACSL3 participated in cardiolipin metabolism.

**Conclusion:** Dehydroabietic acid influences HepG2 cells through the above biological pathways.

## 1 Highlights

1) Proteomics analysis of HepG2 cells after administration of dehydroabietic acid
2) Lipidomics analysis of HepG2 cells after administration of dehydroabietic acid
3) ACSL3 participates in cardiolipin metabolism

## 2 Introduction

Liver cancer is one of the most common cancers worldwide. In recent years, the incidence of liver cancer has shown an increasing tendency each year, becoming a malignant tumor disease with high mortality (Shi et al., 2021). Treatment of liver cancer is limited, and the main effective treatment method is surgical resection supplemented by chemotherapy and radiotherapy. Postoperative tumor recurrence continues to seriously affect the survival of patients (Orcutt and Anaya, 2018; Sun et al., 2021). Therefore, the identification of effective anti-liver cancer drugs has important clinical value and social significance. Dehydroabietic acid is the main compound of rosin, derived from conifers such as spruce, larch and fir (Zhu et al., 2014). Dehydroabietic acid has anti-inflammatory biological activities. For instance, through the dual activation of PPAR-γ and PPAR-α, dehydroabietic acid reduces insulin resistance and hepatic steatosis induced by a high-fat diet (Xie et al., 2020); it reduces iron death in nonalcoholic fatty liver disease by activating the Keap1/Nrf2-ARE signaling pathway (Gao et al., 2021). In the laboratory, we found that dehydroabietic acid can inhibit the growth of liver cancer cells, and we successfully screened out the genes that affect the treatment of dehydroabietic acid in the treatment of liver cancer and the genes related to cell anxiety death, based on which we established a survival line map (Han and Zhang, 2021). The potential objective and pathway of dehydroabietic acid in hepatic carcinoma have rarely been studied, and the potential molecular mechanisms remain unknown. Since the *in vivo* action of dehydroabietic acid is extremely complicated, traditional analytical methods have experienced difficulty disclosing its effective mechanism. With the rapid development of bioinformatics and systematic biology, proteomics is used as a new powerful method to disclose the complicated mechanism of drugs involving biochemical processes of disease progression. It can provide guidance for the early diagnosis, treatment and prognosis of diseases (Monti et al., 2019). Additionally, overall treatment has been one of the most critical clinical strategies, and proteomics can provide multitarget predictions (KhalKhal et al., 2019). Hence, new network science using proteomics can reveal the mechanism of action of dehydroabietic acid in inhibiting hepatic carcinoma. In addition, lipid metabolomics is a technology used to study all intermediate and final products of lipid metabolism in organic bodies and combines bioinformatics analysis. It is characterized by high throughput, high accuracy, high sensitivity, and low cost (Liu and Xu, 2018). Recently, lipid metabolomics has been used to explore effective metabolites after disease treatment by many drugs to describe the molecular process in the mechanism of drug action. It is a special effective method and can screen key metabolic pathways effectively from drugs. In this study, key targets and pathways by which dehydroabietic acid inhibits the proliferation of hepatic carcinoma cells were screened and verified by combining proteomics, lipid metabolomics and bioinformatics analyses. The molecular mechanism of action of dehydroabietic acid in hepatic carcinoma was disclosed. The results provide scientific references to develop dehydroabietic acid as a new drug for hepatic carcinoma.

## 3 Materials

### 3.1 Experimental materials

Dehydroabietic acid standard (batch number: PCS-210816) was purchased from Chengdu Plant Standard Pure Biotechnology Co., Ltd. Methyl alcohol, acetonitrile, methyl tertiary butyl ether, ammonium formate and dichloromethane for LC‒MS were purchased from CNW Technologies, and isopropanol was purchased from Fisher Chemical. Urea, dithiothreitol (DTT), Tris, iodoacetamide (IAA), formic acid and ammonium hydroxide were purchased from Sigma. Trypsin was purchased from Promega. Acetonitrile and water for LC‒MS were purchased from Thermo. A BCA protein concentration assay kit and protease inhibitor were purchased from Beyotime Biotechnology. The ultrafiltration centrifugation column (10 kD) was purchased from PALL (United States). Other reagents were analytically pure and made in China. Fetal calf serum, streptomycin/penicillin and pancreatin were purchased from Gibco. The Lipofectamine™ 3000 transfection reagent was purchased from Thermo Fisher (Article No.: L3000015). The human cardiolipin ELISA kit was purchased from EK Bioscience (Article No.: EK-H11830). The reverse transcription reagents were purchased from Nanjing Novizan Co. LTD (Article No.: R323-01). The real-time quantitative PCR reagent was purchased from Nanjing Novizan Co., Ltd. (Article No.: Q311-03). The internal standard substance was 15:0–18:1-d7-PE is the internal standard substances.

HepG2 cells were purchased from the cell bank of Chinese Academy of Sciences, and dehydroabietic acid (batch No.: 210816) was purchased from Chengdu Purechem-standard Co., Ltd. TFRC antibody (Article No.: ab214039), ACACA antibody (Article No.: Ab45174) and CRLS1 antibody (Article No.: A12388, abclonal) were purchased from Abcam Company. β-actin (A5441) was purchased from Sigma. TRIzol was purchased from ThermoFisher Scientific Co., Ltd. HiScript Q Select RT SuperMix for qPCR and qPCR SYBR Green Master Mix were purchased from Nanjing Vazyme Biotech Co., Ltd.

### 3.2 Experimental apparatus

Vanquish ultrahigh-performance liquid chromatography, Q Exactive HFX high-resolution mass spectrometry and a Heraeus Fresco17 centrifugal machine were purchased from Thermo Fisher Scientific. The BSA124S-CW balance was purchased from Sartorius. The JXFSTPRP-24 grinding mill was purchased from Shanghai Jingxin Technology Co., Ltd. The YM-080S ultrasonic apparatus was purchased from Shenzhen Fangao Microelectronics Co., Ltd. Other experimental apparatuses included the EASY-nLC 1000 liquid chromatogram (Thermo, United States), Thermo Orbitrap Fusion Tribrid mass spectrometer (Thermo, United States), Eppendorf refrigerated centrifuge (Eppendorf, Germany), vacuum freeze drier (Thermo, United States), ultrasonic cell disruptor VCX130 (Sonics, United States), ELISA (MK3 Thermo), and real-time fluorescence quantification PCR instrument (CFX96 Bio-Rad).

The protein electrophoresis system (V140992 Bio-Rad), ECL chemiluminescence detection system (Las-4000 GE), real-time fluorescence quantitative PCR (qPCR) instrument (CFX96 Bio-Rad), and Nanodrop (Nanodrop2000 Thermo) were used.

## 4 Materials and methods

### 4.1 Proteomic and lipidomic cell collection methods

HepG2 cells were divided into 2 groups: the control group and the experimental group (24-h pretreatment with 25 μg/ml dehydroabietic acid), with 3 replicates per group. For the proteome, the suspension cell collection method was adopted; the cells in the logarithmic growth stage were collected into a 15-ml centrifuge tube centrifuged at 1000 g, 4°C, for 5–10 min, and the supernatant was discarded. The cells were washed 3 times with 10 ml PBS. Finally, the cells with 1 ml PBS were resuspended and transferred to a new 1.5-ml centrifuge tube and centrifuged again to remove as much supernatant as possible. Liquid nitrogen was snap-frozen and stored at −80°C for a short period. A previously published report was followed for the lipidomics sample collection method (Sellick et al., 2011).

### 4.2 Proteomics

#### 4.2.1 Protein extraction

Six samples were collected, to which 0.5 ml lysis buffer (8 M urea, 100 mM Tris-HCl pH 7.6, protease inhibitor) was added. After 15 min of ice-bath ultrasound and 15 min of centrifugation at 18000 *g*, the supernatant was collected. Quantitative analysis was carried out using the BCA method. Each sample was collected (20 μg) and mixed in a pool to build a library. Enzymolysis of six samples and pools was implemented by the FASP (filter-aided sample preparation) method.

#### 4.2.2 Enzymatic digestion of proteins

Protein samples (total protein: 100–500 μg) were collected, to which 50 mM DTT was added according to volume for 40 min at 56°C. The ultrafiltration tube was put into the collection pipe, and the previously diluted protein samples were added into the ultrafiltration tube and centrifuged for 15 min at a rate of 12000 g. Next, 200 μl of urea buffer was added to the ultrafiltration tube and then centrifuged for 15 min at 12000 g. Later, 100 μl urea buffer containing 50 mM IAA was added to the ultrafiltration tube and incubated for 20 min in the dark. The mixture was centrifuged for 10 min at 12000 g. Next, 100 μl urea buffer was added and centrifuged for 10 min at 12000 g. This process was repeated twice. Later, 100 μl of 50 mM ABC was added to the ultrafiltration tube and centrifuged for 10 min at 12000 g. Then, 80 μl of ABC with 50 mM pancreatin was added (pancreatin:protein = 1:50–1:100). The mixture was oscillated for 1 min, and the ultrafiltration tube was placed into a water bath at 37°C for 16–18 h. The ultrafiltration tube was transferred to a new collection tube and centrifuged for 10 min at 12000 g. Next, 50 μl of ABC was added to the ultrafiltration tube and centrifuged for 10 min at 12000 g. The protein concentration was tested directly by using the nanodrop protein concentration test mode.

#### 4.2.3 Library construction of data dependence acquisition

One-dimensional high-pH reversed-phase separation of pooled samples was conducted. The merged peptide fragments were dissolved in 100 μl A-phase solution and centrifuged. The supernatant was collected using an injection syringe and injected into the loading ring. The mixture was separated and collected according to a chromatography gradient: the chromatographic column: Gemini-NX 5μ C18 110A 250 × 4.6 mm (Phenomenex, Guangzhou, China); chromatographic apparatus: Shimadzu LC-20AB HPLC Pump system; A phase: 2% CAN, pH 10; B phase: 98% CAN, pH 10; ultraviolet detection wavelength: 214 nm flow rate: 1000 μl/min; chromatography gradient: 0.1–20 min, 5%→30% B; 20–22 min, 30%→80% B; 22–24 min, 80% B; 24–24.1 min, 80%→5% B. Six components were collected and merged according to peak type and time, followed by vacuum centrifugation concentration (rotation vacuum Christ RVC 2–25, Christ, Germany). In the reversed-phase liquid mass coupling RPLC‒MS and DDA analysis, six component samples were collected for LC‒MS/MS testing. Polypeptide samples were dissolved into 25 μL A solution (water containing 0.1% formic acid, with iRT standard peptides). A 5-μl sample solution was loaded into the EASY-nano-LC chromatographic system and onto the precolumn at a flow rate of 4.5 μl/min. Next, the mixture was separated on the analytical column at a flow rate of 300 nL/min. The chromatographic separation gradients were as follows: 0–3 min, B solution (acetonitrile containing 0.1% formic acid) increased linearly from 3% to 7%; 3 min–83 min, B solution increased linearly from 7% to 20%; 83–107 min, B solution increased linearly from 20% to 32% and then increased to 90% over 1 min with holding until 120 min. Mass spectrometric data were acquired using an Orbitrap Fusion mass spectrometer (Thermo Scientific). Specific parameters were set as follows: the spray voltage of the ion source was set to 2.1 kV, the cycling time was set to 4 s, and the level-1 scanning scope was 350–1500 m/z, with a resolution of 60 K (at m/z 200), an AGC target of 4e5, and a maximum IT of 50 ms. The level-2 resolution was 30 K (at m/z 200), with an isolation window of 1.6 Th, AGC target of 5e4, and maximum IT of 120 ms. The MS2 activation was HCD (collision energy: 35).

#### 4.2.4 DIA analysis

Samples of 2 μg peptides each were collected. Appropriate amounts of iRT standard peptides were mixed into each sample, and each sample was subjected to DIA mass spectrometry once for 2 h.

#### 4.2.5 Peptide data statistics

DDA original data collected by mass spectrometry were input into Spectronout Pulsar X (Biognosys Company) for library search analysis. The human protein dataset downloaded from UniProt was used. Parameters for building the library used the default optimal parameters “GBS factory setting”, including a tolerance of MS1 = 10 ppm, tolerance of level-2 pieces = 0.02 Da, and maximum allowable missing number = 2. Cysteine carbamidomethyl was a fixed modification, while oxidization of methionine and N-terminal acetylation of protein were variable modifications. The false-positive rate of the peptide and protein levels was FDR<0.01, and the independent peptide was at least 1. Next, the DIA original data were input into Spectronout Pulsar X for qualitative and quantitative protein analysis. Parameters for building the library were introduced as follows: Peptides FDR PSMs FDR Proteins FDR = 1%. At least three peptides were chosen for each type, and at most 6 optimal subions were chosen to produce the library spectra: iRT Calibration Rsquare>0.8. Quantitative parameter: The iTR standard curve used local (nonlinear) regression. Protein identification utilized a precursor Q_value_ cutoff = 0.01 and protein Q_value_ cutoff = 0.01. A kernel density estimator was applied for *p*-value calibration. Protein quantification used the subion peak area, and the average strength quantification of at least 3 subions was chosen.

#### 4.2.6 Differential protein expression

Statistical analysis of protein expression data is needed after completing protein expression quantification. Significantly differentially expressed proteins under different states were screened. The fold changes in protein in different comparison groups were calculated, and significance was examined by the Welch’s t test. Therefore, significantly expressed proteins (FC ≥ 1.5 且 *p* ≤ 0.05) were screened. Finally, enrichment analysis of different proteins was performed.

### 4.3 Nontarget lipidomics experiment

#### 4.3.1 Extraction of metabolites

Water (400 μl) was added to the samples and mixed uniformly in a vortex for 30 s. The mixture was placed in a liquid nitrogen tank and frozen for 1 min. Later, the mixture was removed and thawed. These procedures were repeated 2–3 times, followed by 10 min of ice water bath ultrasound. Next, 960 μl extraction solution (MTBE: MEOH = 5:1, containing IS) was added to the mixture and then mixed uniformly in a vortex for 30 s, followed by 10 min of ice water bath ultrasound. The mixture was kept static for 1 h at −40°C. Samples were centrifuged for 15 min at 4°C at a rate of 3000 rpm (centrifugal force of 900 ×*g*). The supernatant (600 μl) was collected and placed in an EP tube, dried under vacuum, and added to 200 μl of solution (DCM:MeOH = 1:1) for redissolving. The mixture was mixed by vortexing for 30 s, followed by 10 min of ice water bath ultrasound. Samples were centrifuged for 15 min at 4°C at a rate of 13000 rpm (centrifugal force 16200 ×*g*). A total of 75 μl supernatant was collected into a loading bottle for testing.

#### 4.3.2 LC‒MS/MS analysis

In this study, the chromatograph of target compounds was implemented by using a Vanquish (Thermo Fisher Scientific) ultrahigh-performance liquid chromatograph and Waters ACQUITY UPLC HSST3 (2.1 × 100 mm, 1.8 μm) liquid chromatograph. The liquid chromatogram A-phase consisted of a solution containing 40% water and 60% acetonitrile, which also contained 10 mmol/L ammonium formate. The B-phase consisted of a solution containing 10% acetonitrile and 90% isopropanol. A total of 50 ml of a 10 mmol/L ammonium formate aqueous solution was added to the B-phase every 1000 ml. Gradient elution was applied: 0–1.0 min, 40% B; 1.0–12.0 min, 40%–100% B; 12.0–13.5 min, 100% B; 13.5–13.7 min, 100%–40% B; 13.7–18.0 min, 40% B. The flow rate of the moving phase was 0.3 ml/min, the column temperature was 55°C, the sample plate temperature was 4°C, and the sampling volume was 2 μL for positive ions and 2 μl for negative ions.

Thermo Q Exactive HFX mass spectrometry could achieve level-1 and level-2 mass spectrometric data acquisition under the management of the controlling software (Xcalibur, Version 4.0.27, Thermo). Detailed parameters were introduced as follows: sheath gas flow rate of 30 Arb, aux gas flow rate of 10 Arb, capillary temperature of 350°C, full MS resolution of 120000, MS/MS resolution of 7500, collision energy of 10/30/60 in NCE mode, and spray voltage of 4 kV (positive) or −3.8 kV (negative).

#### 4.3.3 Data processing

The original format of mass spectra was transformed into the mzXML format using ProteoWizard. Next, XCMS was applied for retention time correction, peak recognition, peak extraction, peak integral and peak alignment. Minfrac and cutoff were set to 0.5 and 0.3, respectively. In this experiment, lipids were identified by using XCMS software, the self-writing R program package and the lipidblast database. SIMCA-P 14.1 software was applied for bioinformatics and statistical analyses.

### 4.4 Omics data conjoint analysis

Differentially expressed genes of ferroptosis and differentially changing lipid molecules were screened by using HepG2 cells under the administration of dehydroabietic acid. Conjoint analysis of relevant data was carried out, and ggPlot 2 using R packets with the Spearman statistical method was applied.

### 4.5 ACSL3 participates in cardiolipin metabolism

#### 4.5.1 Cell culture

HepG2 cells were cultured in high-glucose DMEM containing 10% FBS, 100 U/mL penbritin and 100 μg/ml streptomycin. The conditions of the incubator were 37°C and 5% CO_2_.

#### 4.5.2 Construction of ACSL3 knockdown cell lines

HepG2 cells were inoculated into 6-well plates at a density of 4×10^4^ cells/mL and then cultured overnight until they adhered to the walls. On the second day, siNC and siACSL3 were transfected into cells using Lipofectamine™ 3000 transfection reagent. Specific experimental steps and dosages referred to the specification. After transfection for 6 h, the medium was changed to fresh complete culture media to culture cells continuously. After transfection for 24 h, cell samples were collected and tested. The siRNA sequences were as follows:

siACSL3-1-FP: 5′-GCC​CUC​AGA​UAU​UGC​AGU​AAU-3′,

siACSL3-1-RP: 5′-AUU​ACU​GCA​AUA​UCU​GAG​GGC-3′;

siACSL3-2-FP: 5′-GCG​GAC​AUU​GAG​CGA​AUG​UAU-3′,

siACSL3-2-RP: 5′-AUA​CAU​UCG​CUC​AAU​GUC​CGC-3′;

siACSL3-3-FP: 5′-CCU​GGA​UGU​GAU​ACU​UUA​GAU-3′,

siACSL3-3-RP: 5′-AUC​UAA​AGU​AUC​ACA​UCC​AGG-3′.

#### 4.5.3 Real-time quantitative PCR experiment

After RNA extraction, a reverse transcription experiment was carried out according to the manufacturer’s instructions. The RNA volume for reverse transcription was 1 μg. The reverse transcription cDNA was added to an appropriate volume of ddH_2_O and then diluted twice. Real-time florescence quantitative PCR of reverse transcription cDNA was carried out with reference to the specifications of reagents from Nanjing Novizan Co., Ltd. The primer sequences were as follows:

ACSL3-F: TCC​TGT​TGG​TCA​GGG​ATA​CG;

ACSL3-R: ATC​CAC​CTT​CCT​CCC​AGT​TT.

GAPDH-F: GGA​CTC​ATG​ACC​ACA​GTC​CA;

GAPDH-R: TCA​GCT​CAG​GGA​TGA​CCT​TG.

#### 4.5.4 Enzyme-linked immunosorbent assay

Cell supernatants and broken cell lysis buffer of the siNC group and siACSL3 group were collected. The cardiolipin content in these cells was tested using a Human Cardiolipin ELISA kit with reference to the specifications.

### 4.6 CCK8 cell proliferation test

Dehydroabietic acid was fully weighed and dissolved in DMSO to prepare a 10 mg/ml stock solution. Dehydroabietic acid was diluted to final concentrations (0, 3.125, 6.25, 12.5, 25, 50, 100, 200, 400, and 800 μg/ml) with complete medium. DMSO was used as the control group.

Cells in the logarithmic phase were inoculated into a 96-well plate, with 5000 cells per hole. The 96-well plate was cultivated in an incubator overnight, and then the culture medium was removed. Media with different concentrations of drugs were added to each experimental group and cultivated for 24 h continuously. Three parallel wells were created for each group. DMSO of the corresponding volume was added to the media and used as the blank control group. After treatment, 10% CCK8 solution (10 μlCCK8/100 μl media) was added to each well. Cells were cultivated in an incubator at 37°C for 1 h, and the absorbance at 450 nm was determined by ELISA.

### 4.7 Bioinformatics analysis of ferroptosis-related genes

#### 4.7.1 Protein‒protein interaction network analysis

Differential proteins were input into the STRING (https://string-db.org/) database for protein‒protein interaction (PPI) analysis. The confidence interval was chosen as 0.4, and visual analysis was carried out using Cytoscape software. The top 6 genes were recognized by the Degree algorithm of the CytoHubba plug-in in Cytoscape, and they were defined as the Hub genes in the present study.

#### 4.7.2 Differential expression analysis of hub genes in hepatic carcinoma and normal tissues

The hepatic carcinoma (HCC) RNA-Seq dataset was downloaded from the TCGA database. Later, the differential expression of hub genes at the transcriptional level in normal tissues and hepatic carcinoma tissues was analyzed by two-sample t-tests.

#### 4.7.3 Hub gene expression and clinicopathologic data analysis

Clinical data of patients with HCC were downloaded from the TCGA database. The inclusion criteria were as follows: 1) patients with primary HCC (recurrent HCC excluded); 2) complete clinicopathologic features; 3) available RNA-seq data; and 4) OS set as the major endpoint. The exclusion criteria were as follows: 1) patients who had HCC recurrence according to pathological diagnosis; 2) patients with tumors other than HCC; and 3) missing survival information and clinical pathological parameters. First, patients were divided into a high-expression group and a low-expression group according to the medians of Hub gene expression and assessed by the Kaplan‒Meier survival map. Additionally, the relationships of Hub gene expression with the survival and prognosis of patients with HCC were analyzed by the 95% confidence interval of the hazard ratio (HR) and based on the *p*-value of the log-rank test (log-rank P). Furthermore, the differential expression of hub genes in different pathological stages was analyzed.

#### 4.7.4 Single-gene GSEA

The hallmark gene set in the MSigDB on the GSEA (https://www.gsea-msigdb.org/gsea/index.jsp) website was used as the reference gene set. The potential effect pathways of Hub genes in HCC development were analyzed by the default weighted enrichment statistical method. RNA-seq data samples of patients with HCC were divided into high-expression and low-expression groups according to medians of Hub gene expression. The pathways with an adjusted *p*-value < 0.05, FDR<0.25 and NES absolute ≥1 were considered significantly enriched.

### 4.8 Molecular docking of dehydroabietic acid with CRLS1, ACACA and TFRC

CRLS1 is one of the key enzymes in the synthesis of cardiolipin, and it can accelerate the energy consumption of adipocytes (Sustarsic et al., 2018). Acetyl-CoA carboxylase (ACC) is a key enzyme in fatty acid synthesis (Chen et al., 2019). TFRC can bind with transferrin, which carries free iron and is the key protein that participates in the formation of ferroptosis (Jiang et al., 2021). The 3D structures of 3 proteins were acquired from UniProt: ACC: https://www.uniprot.org/uniprotkb/Q13085/entry#structure TFRC: https://www.uniprot.org/uniprotkb/P02786/entry#structure CRLS1: https://www.uniprot.org/uniprotkb/Q9UJA2/entry#structure. The 3D structure of dehydroabietic acid was acquired by chem3D. Open the protein 3D model in AutoDocktools for preprocessing, such as hydrogenation and charge calculation. The substrate molecules were built by Chemdraw and stored in SDF format. Dehydroabietic acid preprocessing was carried out in Atudodocktools. The docking grid documents were generated by AutoGrid of sitemap, and AutoDock was used for docking. A total of 10 docking results were produced. The docking results with the optimal conformation were chosen. PyMOL 1.6.5 software was chosen for analysis, and diagrams were plotted.

### 4.9 Protein expression analysis of CRLS1, ACACA and TFRC

In this experiment, immunohistochemical images of ACACA and TFRC in normal tissues and HCC tissues were downloaded from the human protein atlas HPA database (http://www.proteinatlas.org), which publicly available. These images were used to compare the expression differences of ACACA and TFRC in HCC and normal liver tissues at the translational level. No expression differences in CRLS1 at the translation level between HCC and normal tissues were found.

### 4.10 Reverse transcription and real-time qPCR

Reverse transcription reactions were carried out according to the kit supplied by Nanjing Vazyme Biotech Co., Ltd. The reverse transcription RNA quantity was 1 μg. Real-time qPCR was implemented with reference to the kit supplied by Nanjing Vazyme Biotech Co., LTD. The reaction solution was prepared according to Table 1.

**TABLE 1:** RT‒PCR system.

Reagent	Volume (μL)
SYBR Green I (2*)	10
primer F (10 μM)	1
primer R (10 μM)	1
Addition of ddH2O	20

After the reaction liquid was prepared, the liquid was successively added into the 96-well plate followed by the cDNA. Next, reactions were carried out on the real-time qPCR instrument according to the instructions. Each gene was tested 3 times using specific primers. The expression level of genes was determined with GAPDH as the internal reference. The relative quantitative expression of genes was provided by the 2^-△△ Ct^ method. The primer sequences are shown in Table 2.

**TABLE 2:** Primer sequences for real-time qPCR.

Primer names	Primer sequence (5′-3′)
CRLS1-homo-qF	CCG​AAC​TCT​TCC​AAC​ACC​AC
CRLS1-homo-qR	CAA​AGA​AGC​TGC​CAC​CAA​GA
ACACA-homo-qF	ATA​AGG​ATC​TGG​CGG​AGT​GG
ACACA-homo-qR	TCC​ATG​GCA​ACC​TCT​GGA​TT
GAPDH-homo-qF	GGA​CTC​ATG​ACC​ACA​GTC​CA
GAPDH-homo-qR	TCA​GCT​CAG​GGA​TGA​CCT​TG

### 4.11 Western blot assay

Equivalent amounts of protein were loaded into SDS‒PAGE gel pores for 2 h of electrophoresis at 100 V. Membrane transformation was performed for 1 h after the completion of electrophoresis under a constant current of 200 mA. Later, the membrane was closed with 5% closed solution for 1 h at room temperature and then incubated in 5% closed solution at room temperature for 2 h using the first antibody at an appropriate dilution ratio. Next, the membrane was washed 3 times with TBST for 5 min each. Later, the membrane was incubated in TBST with 5% closed buffer solution for 1 h at room temperature using the marked second antibody with a dilution ratio of HRP, followed by 3 washes with TBST for 5 min each. Finally, hypersensitivity ECL chemiluminescent reagent was added to the PVDF membrane for exposure imaging and photography. β-actin was used as the internal reference, and the relative protein expression level of the bands was analyzed using Quantity One software.

## 5 Results

### 5.1 Proteomics analysis of HepG2 cells after administration of dehydroabietic acid

#### 5.1.1 Quantitative analysis of proteins

In this experiment, preset test groups were tested and calculated using Welch’s t test. According to the analysis, 1221 differentially expressed proteins (FC ≥ 1.5 and *p* ≤ 0.05) were screened from the test groups and the control group, including 260 upregulated and 961 downregulated proteins.

#### 5.1.2 Gene ontology enrichment analysis

**FIGURE 1:**
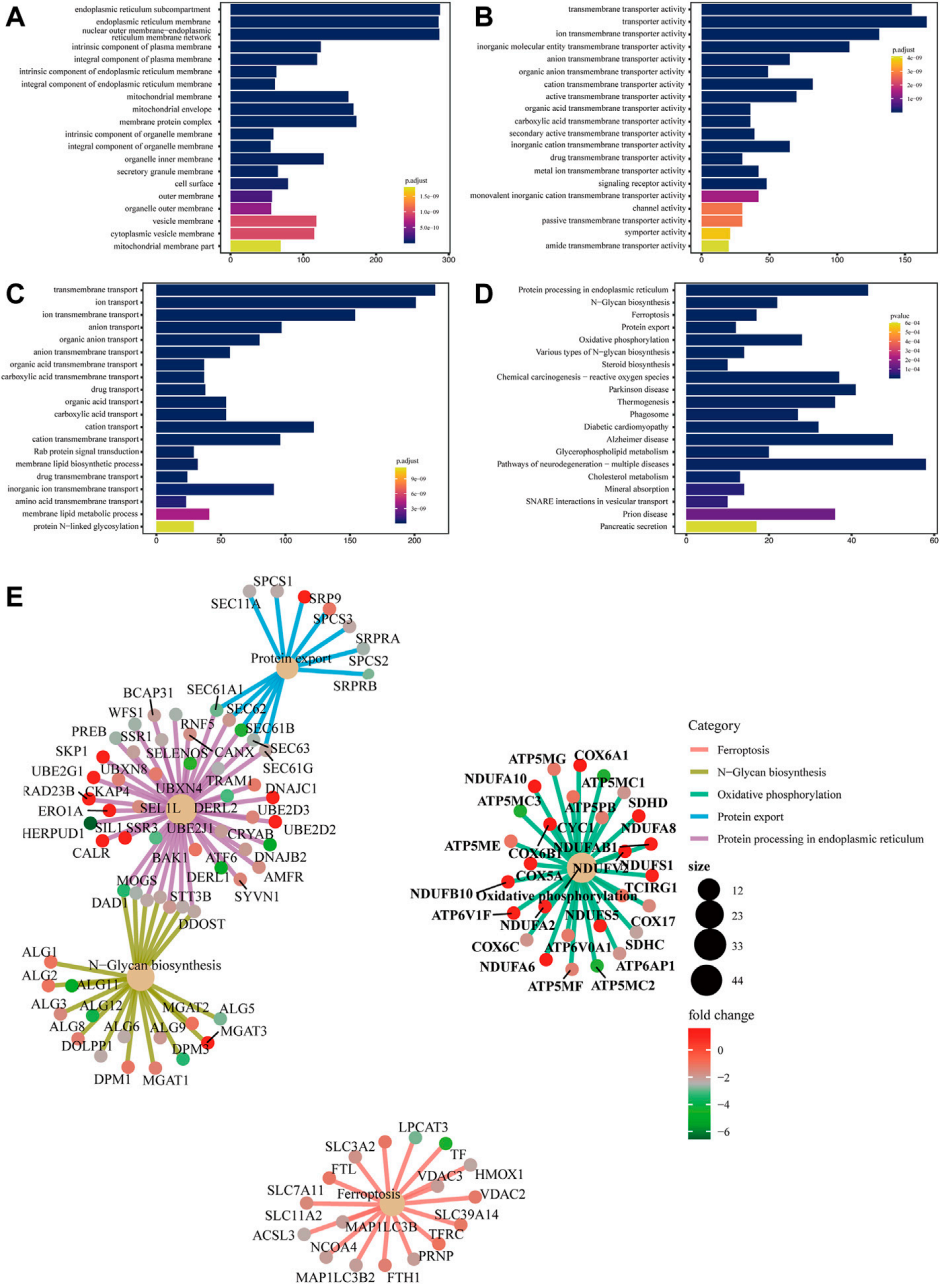
Bar chart of GO enrichment analysis and bar chart and network diagram of pathway enrichment analysis **(A)** Bar chart of CC enrichment analysis. **(B)** Bar chart of MF enrichment analysis. **(C)** Bar chart of BP enrichment analysis. The x-axis shows the protein quantity, and the y-axis shows the GO term. Colors correspond to statistical significance and show the top 20 GO terms in terms of enrichment significance. **(D)** Bar chart of pathway enrichment analysis. The x-axis shows the protein quantity, and the y-axis indicates the pathway. Colors correspond to statistical significance and show the top 20 pathways in terms of enrichment significance. **(E)** Network diagram of pathway enrichment analysis. The shallow yellow circles are pathways, and the small dots are proteins. Representative proteins of the pathway are shown. The y-axis indicates the pathway. The circle size denotes the number of enriched proteins, and colors correspond to statistical significance.

The main function of the GO database is to provide annotation of protein functions. It has three major branches: cellular component (CC), molecular function (MF) and biological process (BP). The terms in CC were used to describe the positions of protein products inside and outside of cells. Terms in MF were used to define functions on the molecular layers of protein products. Terms in BP were used to describe biological paths or mechanisms in which protein products participate. CC, MF, and BP were mainly enriched in endoplasmic reticulum membrane (Figure 1A), transporter activity (Figure 1B) and transmembrane transport (Figure 1C), respectively.

#### 5.1.3 Pathway enrichment analysis

Biological pathways in which the differentially expressed proteins were located were analyzed. The results showed that metabolic pathways with significant enrichment of differential proteins (*p* < 0.05) mainly participated in ferroptosis, N−glycan biosynthesis, and oxidative phosphorylation, among others (Figure 1D). A network diagram of the top five pathways in terms of enrichment significance was built based on the common enriched proteins, which was conducive to discovering the relationship between proteins and pathways. Ferroptosis-enriched proteins included ACSL3, TFRC, and FTH1 (Figure 1E).

### 5.2 Lipidomics analysis of HepG2 cells after administration of dehydroabietic acid

#### 5.2.1 PCA of samples

**FIGURE 2:**
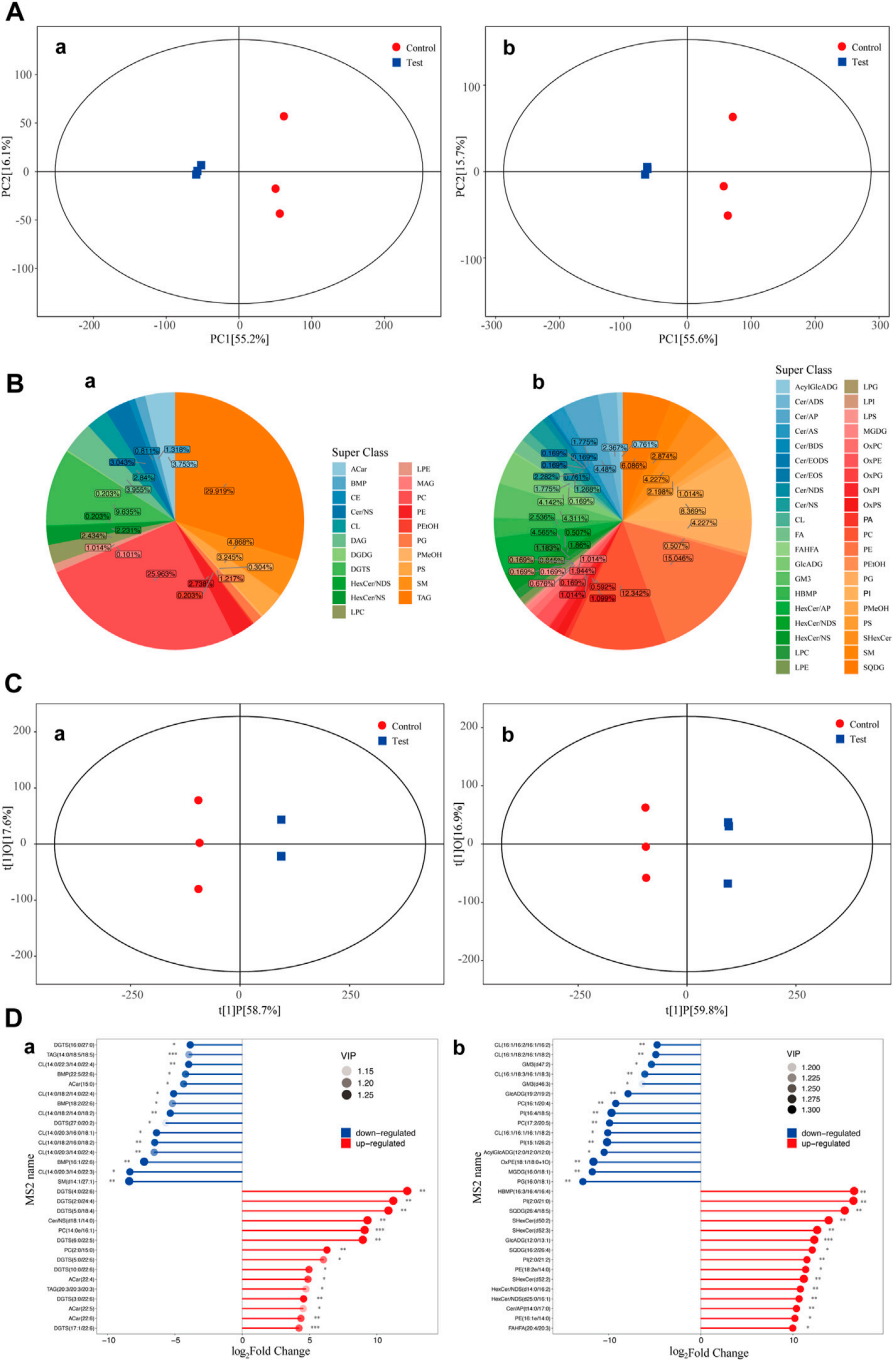
PCA scores of the test group and control group. Diagram of metabolite classification and proportion of the OPLS-DA scores and match stick diagrams **(A)** PCA scores of the test and control groups. X-axis PC [1] and y-axis PC [2] are scores of the first and second principal components. Each scattering point represents a sample. Colors and shapes of scattering points represent different groups. If sample points are distributed more closely, the class and content of metabolites in samples are more similar. In contrast, if sample points are far away from each other, the overall metabolism level differs greatly. **(B)** Pie chart of the classification and proportion of metabolites. Different colors represent different classes, and the percentage refers to the proportion of one metabolite class in the total metabolites. **(C)** OPLS-DA score diagram of the test and control groups. The x-axis t [1]P refers to the predicted score of the first principal component and shows intergroup differences of samples. The y-axis t [1]0 refers to the scores of the orthogonal principal component and shows the intragroup difference of samples. Each scattering point represents a sample. The shapes and colors of the scattering points reflect different test groups. A longer transverse distance among samples indicates greater intergroup differences, and a shorter longitudinal distance reflects better intragroup repeatability. **(D)** Match stick diagrams of the test group vs. control group. The x-axis shows fold changes after logarithmic transformation. The color intensity of the points represents VIP values. Notes: * 0.01 < *p* < 0.05, ** 0.001 < *p* < 0.01 and ***p < 0.001. (a) positive ion mode; (b) negative ion mode.

The overall distribution of all samples was observed by PCA to recognize discrete points. PCA of the test and control groups was carried out using SIMCA-P software. Two PCA models with two principal components were constructed. The PCA scores are shown in Figure 2A. Figure 2A shows that in the positive ion and negative ion modes, there was obvious separation between samples of the test and control groups. PCA model parameters were obtained through 6 cyclic interaction verifications. The interpretation rates of the positive and negative ion models were R^2^X = 0.822 and R^2^X = 0.816, respectively. Hence, it can be concluded that the PCA model could effectively interpret metabolic differences between samples of the test and control groups.

#### 5.2.2 Metabolite classification statistics

Classification statistics of identified metabolites were conducted according to chemical classification information. The proportions of different types of metabolites are shown in Figure 2B.

#### 5.2.3 OPLS-DA of samples

OPLS-DA of the test and control groups was carried out using SIMCA-P software. An OPLS-DA model was built (Figure 2C). Samples of the test group and control group were two-sided, indicating significant differences between the two groups. The model evaluation parameters gained from 6 cyclic interaction verifications were R^2^X, R^2^Y, and Q^2^. The positive ion mode was R^2^X = 0.768, R^2^Y = 1, and Q^2^ = 0.987. The negative ion mode was R^2^X = 0.763, R^2^Y = 1, and Q^2^ = 0.985. These findings indicated that the model was stable and reliable. Moreover, the replacement test of OPLS-DA mode was performed based on the data from the positive and negative ion modes. It could be concluded that no fitting had occurred in the OPLS-DA model, which was built based on data from positive and negative ion models.

#### 5.2.4 Significant differential metabolites

Differential metabolites among groups (VIP>1) were screened according to the variable weight value (VIP). Significant differential metabolites were screened by using single-variable statistical analysis (VIP>1 and *p* < 0.05). A total of 30 significant differential metabolites were screened and identified through statistical analysis (Figure 2D).

#### 5.2.5 Hierarchical cluster analysis of differential metabolites

The screened differential metabolites had similar/complementary biological results and functions or were subordinate to positive/negative regulation by the same metabolic pathway. They showed similar or opposite expression features among different test groups. Hierarchical cluster analysis of these features was conducive to classifying metabolites with the same features together and recognizing variation features of metabolites among test groups.

**FIGURE 3:**
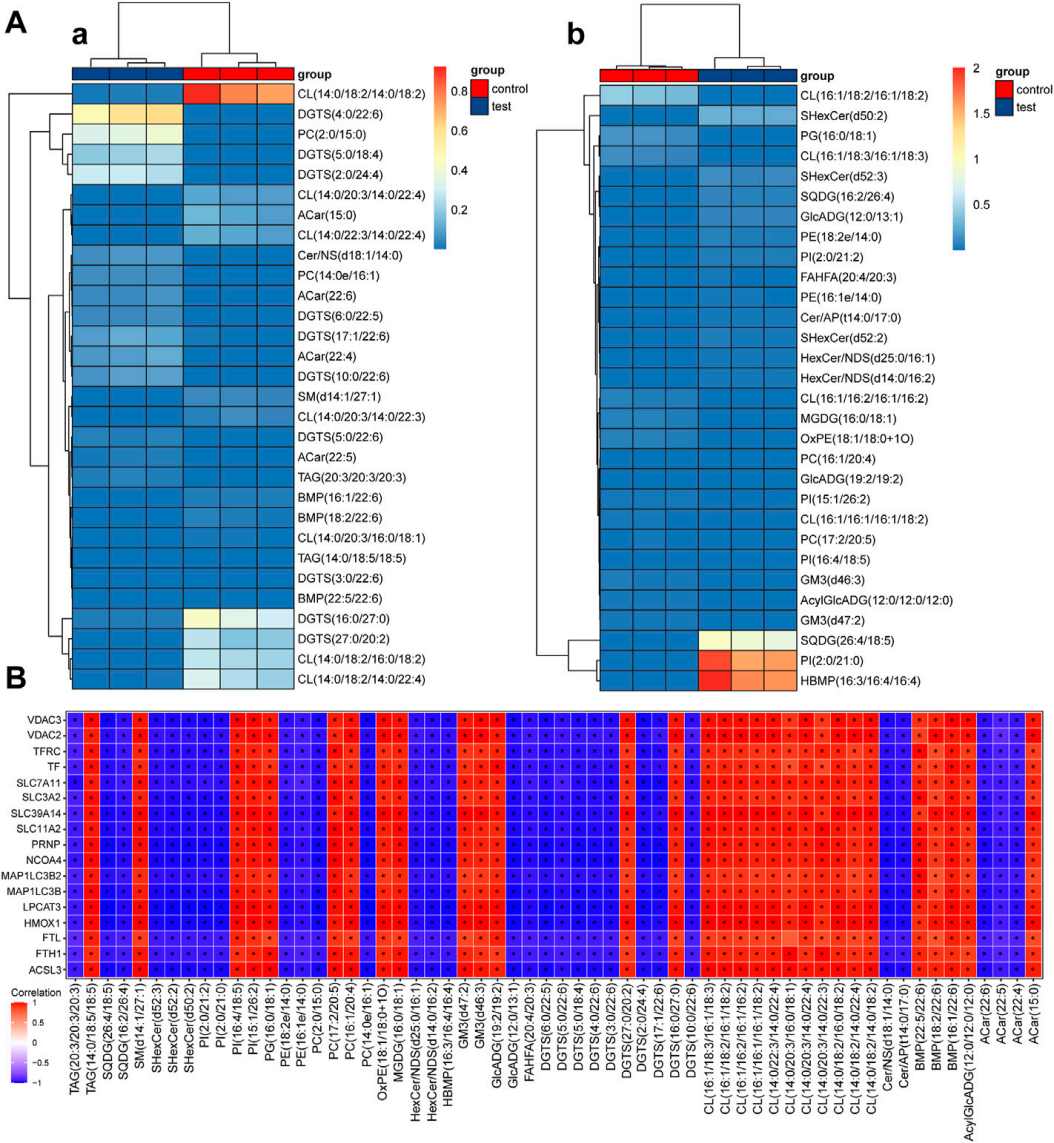
Thermodynamic diagram of the hierarchical clustering analysis of significantly differential metabolites and thermodynamic diagram of correlation between ferroptosis differential proteins and metabolites **(A)** Thermodynamic diagram of hierarchical clustering analysis of significantly differential metabolites between test groups and the control group (a). positive ion mode; (b): negative ion mode. The x-axis represents different test groups, and the y-axis represents differential metabolites of the group. Blocks at different positions represent the relative expression of metabolites at corresponding positions. **(B)** Thermodynamic diagram of the correlation between ferroptosis differential proteins and metabolites. Red (corr = 1), blue (corr = -1) and white (corr = 0). Data with *p* < 0.05 are marked by “*” in the diagram. The x-axis shows the differentially abundant proteins identified by proteomics, and the y-axis shows the differentially abundant metabolites identified by metabolomics.

Hierarchical cluster analysis results of differential metabolites under positive and negative ion modes are shown in Figure 3A. The test group clearly showed significantly downregulated metabolism capacity of CL (14:0/18:2/14:0/18:2), DGTS (16:0/27:0), DGTS (27:0/20:2), CL (14:0/18:2/16:0/18:2), and CL (14:0/18:2/14:0/22:4) compared with the control group. Among them, CL (14:0/18:2/14:0/18:2) was the most significant. Additionally, the metabolic capacity of DGTS (4:0/22:6), PC(2:0/15:0), DGTS (5:0/18:4) and DGTS (2:0/24:4) in the test group was obviously upregulated compared with that in the control group. According to the clustering heatmap, significantly differential metabolites were mainly divided into two classes. The metabolic capacities of SQDG (26:4/18:5), PI(2:0/21:0), and HBMP(16:3/16:4/16:4) in the test group were obviously upregulated compared with those in the control group.

### 5.3 Correlation analysis of ferroptosis differential proteins and metabolites

Correlation coefficients between ferroptosis differential proteins and metabolites were calculated by the Pearson method. The results are shown in a thermodynamic diagram. A good correlation between ACSL3 protein and cardiolipin was found. The results are shown in Figure 3B.

### 5.4 ACSL3 participates in cardiolipin metabolism

#### 5.4.1 Successful establishment of ACSL3 knockdown cell lines

**FIGURE 4:**
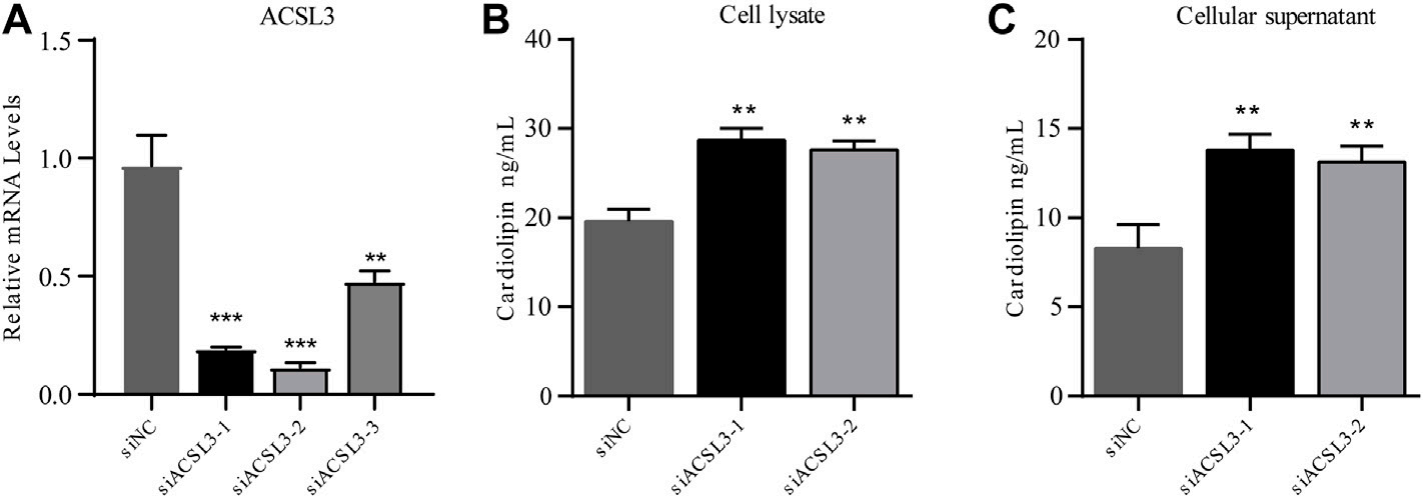
ACSL3 level in ACSL3 knockdown cells and ACSL3 level in cell lysis buffer and supernatant **(A)** The ACSL3 level in ACSL3 knockdown cells. Real-time quantitative PCR analysis of ACSL3 mRNA levels in HepG2 cells after siRNA transfection. ** represents *p* < 0.01. *** indicates *p* < 0.001. **(B)** ACSL3 levels in cell lysis buffer. ELISA results for the cardiolipin content in cell lysis buffer of siNC and siACSL3 HepG2 cells. ** indicates *p* < 0.01. **(C)** ACSL3 levels in the cell supernatant. ELISA analysis of cardiolipin content in siNC and siACSL3 HepG2 cells. ** represents *p* < 0.01.

After HepG2 cells were transfected with siRNA for 24 h, cell samples were collected, and RNA was extracted. The mRNA level of ACSL3 was tested by real-time quantitative PCR. The results demonstrated that all three siRNAs significantly inhibited the mRNA levels of ACSL3. In siACSL3-1 and siACSL3-2, the mRNA level of ACSL3 decreased by approximately 80%, while the knockdown effect of siACSL3-3 was approximately 60% (Figure 4A). Therefore, siACSL3-1 and siACSL3-2 were applied in the follow-up experiment.

#### 5.4.2 ACSL3 knockdown facilitates accumulation of cardiolipin in cell supernatant and lysis buffer

To investigate the regulatory effect of ACSL3 on cardiolipin, the cell lysis buffer and supernatant of the siNC group and siACSL3 group were collected, and the cardiolipin level was tested using an ELISA kit. The results showed that cardiolipin levels in cell lysis buffer and cell supernatant after ACSL3 knockdown increased significantly compared with the control group (Figures 4B,C). Altogether, these results implied that ACSL3 participated in the regulation of cardiolipin metabolism.

### 5.5 Lipidomics and proteomics analysis of molecular models of the effects of dehydroabietic acid on HepG2 hepatocellular cancer cells

To establish a molecular model under the influence of dehydroabietic acid on HepG2 hepatocellular carcinoma cells, we analyzed lipidomics and proteomics and selected metabolic and protein pathways as carriers to map differentially expressed proteins and metabolites. The results showed that the pathways involved in these differentially expressed proteins and metabolites mainly involved iron death and lipid metabolism, indicating that the differentially expressed proteins and metabolites were directly related to the effect of dehydroabietic acid on HepG2 in hepatocellular carcinoma cells.

**FIGURE 5:**
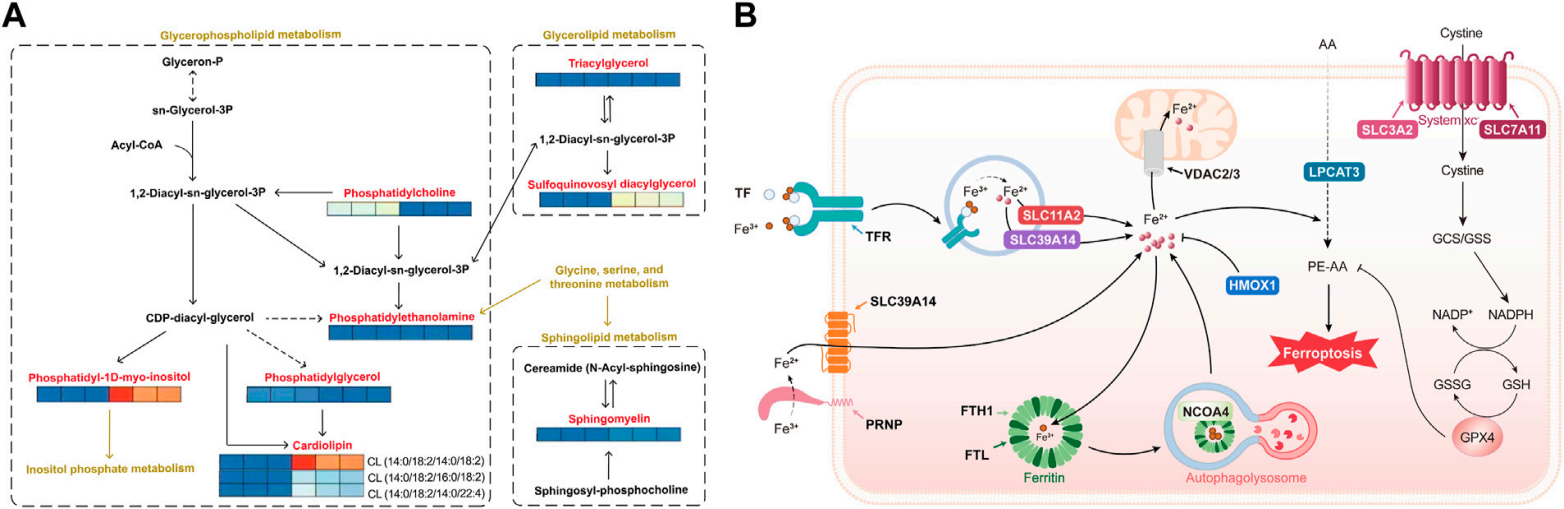
Lipidomics of molecular models of the effects of dehydroabietic acid on HepG2 hepatocellular cancer cells **(A)** and proteomics analysis of molecular models of the effects of dehydroabietic acid on HepG2 hepatocellular cancer cells **(B)**.

Metabolic mapping of lipid metabolism pathways indicated that dehydroabietic acid was quite active in the performance of metabolites affected by HepG2 in hepatocellular carcinoma cells. As shown in Figure 5A, dehydroabietic acid had a significant effect on multiple metabolites in glycerol and phospholipid metabolism, including cardiolipin (CL), phosphatidylcholine (PC) downregulation, sphingomyelin (SM) upregulation, sulfoquinovosyl diacylglycerol (SQDG) upregulation, and phosphatidylinositol (PI) upregulation, among others. Phosphatidylethanolamine (PE), triacylglycerol (TAG) and phosphatidylglycerol (PG) had no significant effects. Notably, dehydroabietic acid significantly upregulated CL (14:0/18:2/14:0/18:2) but had inhibitory effects on CL (14:0/18:2/16:0/18:2) and CL (14:0/18:2/14:0/22:4). In addition, dehydroabietic acid was able to “upregulate” SQDG, an intermediate product of the glyceride metabolic pathway.

Pathway mapping of iron death pathways showed that dehydroabietic acid was quite active in protein performance under the influence of HepG2 in hepatocellular carcinoma cells. As shown in Figure 5B, AA (arachidonic acid) entered the cell and underwent catalysis by ferrous ions and multiple enzymes (including LPCAT3) to produce the toxic substance PE-AA, resulting in iron death. The trivalent iron ions outside the cell were reduced to divalent iron ions under the action of the PRNP protein, and Fe2+ traversed the SLC39A14 protein into the cell interior. In addition, extracellular Fe3+ was able to bind to TF, which was recognized by TFR receptors and enters the cell in the form of vesicles. It then traversed the vesicle transmembrane proteins SLC11A2 and SLC39A14 into the cytoplasm. Part of the Fe2+ in the cytoplasm entered the mitochondria through the mitochondrial membrane protein VDAC2 or 3 to function, and the other part in the form of Fe3+ was stored in ferritin composed of FTH1 (heavy chain) and FTL (light chain). This portion of Fe3+ was self-delivered to autophagic lysosomes under the action of NCOA4, resulting in Fe2+ production. SLC3A2 and SLC7A11 together formed the system xc, which transferred cystine from outside the cell to the intracellular space, thereby promoting GSH production and affecting GPX4 activity. GPX4 was effective in clearing PE-AA and inhibiting iron death. Here, we found that dehydrorosin could inhibit the protein indicated in bold text in the figure, thereby preventing iron death.

### 5.6 Dehydroabietic acid obviously inhibits the growth of hepatic carcinoma cells

**FIGURE 6:**
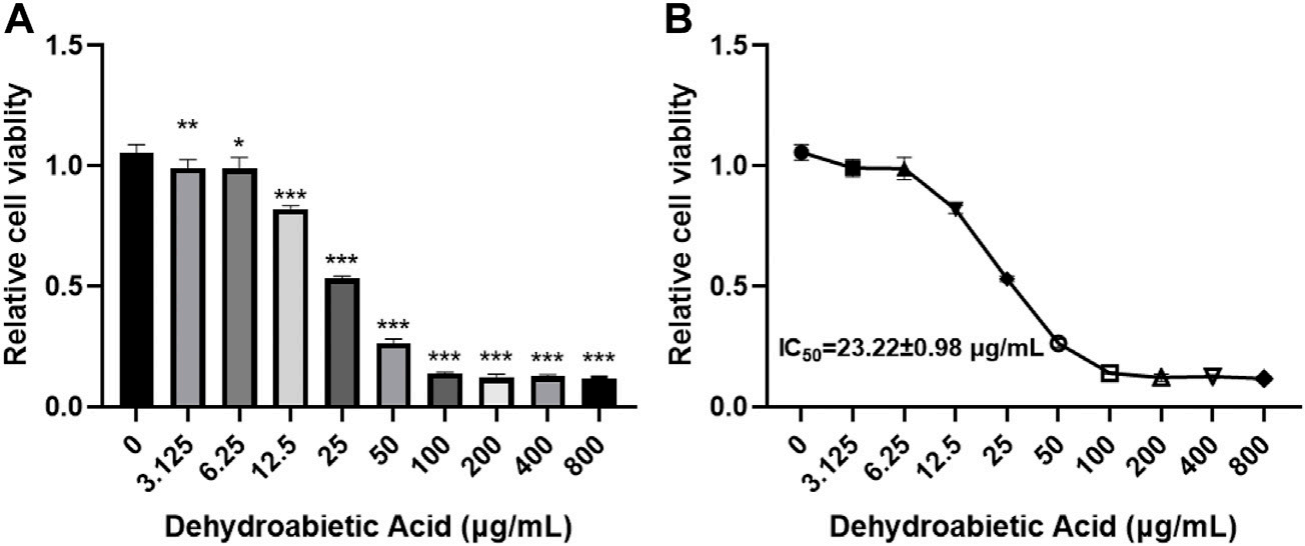
Effects of dehydroabietic acid on the growth of HCC cells **(A)**: CCK8 analysis of the effects of dehydroabietic acid at different concentrations on cell growth. **(B)**: IC50 value of dehydroabietic acid.

In this study, different concentrations of dehydroabietic acid were applied to process HepG2 cells for 72 h. According to the CCK8 results, dehydroabietic acid obviously inhibited cell growth at concentrations from 3.125–800 μg/ml in a concentration-dependent manner (Figures 6A,B). According to further results, the IC50 value of dehydroabietic acid was 23.22 ± 0.98 μg/ml. Hence, cells were treated with this concentration in the follow-up experiment.

### 5.7 Bioinformatics analysis of ferroptosis-related genes

#### 5.7.1 Hub gene screening in protein‒protein interaction

**FIGURE 7:**
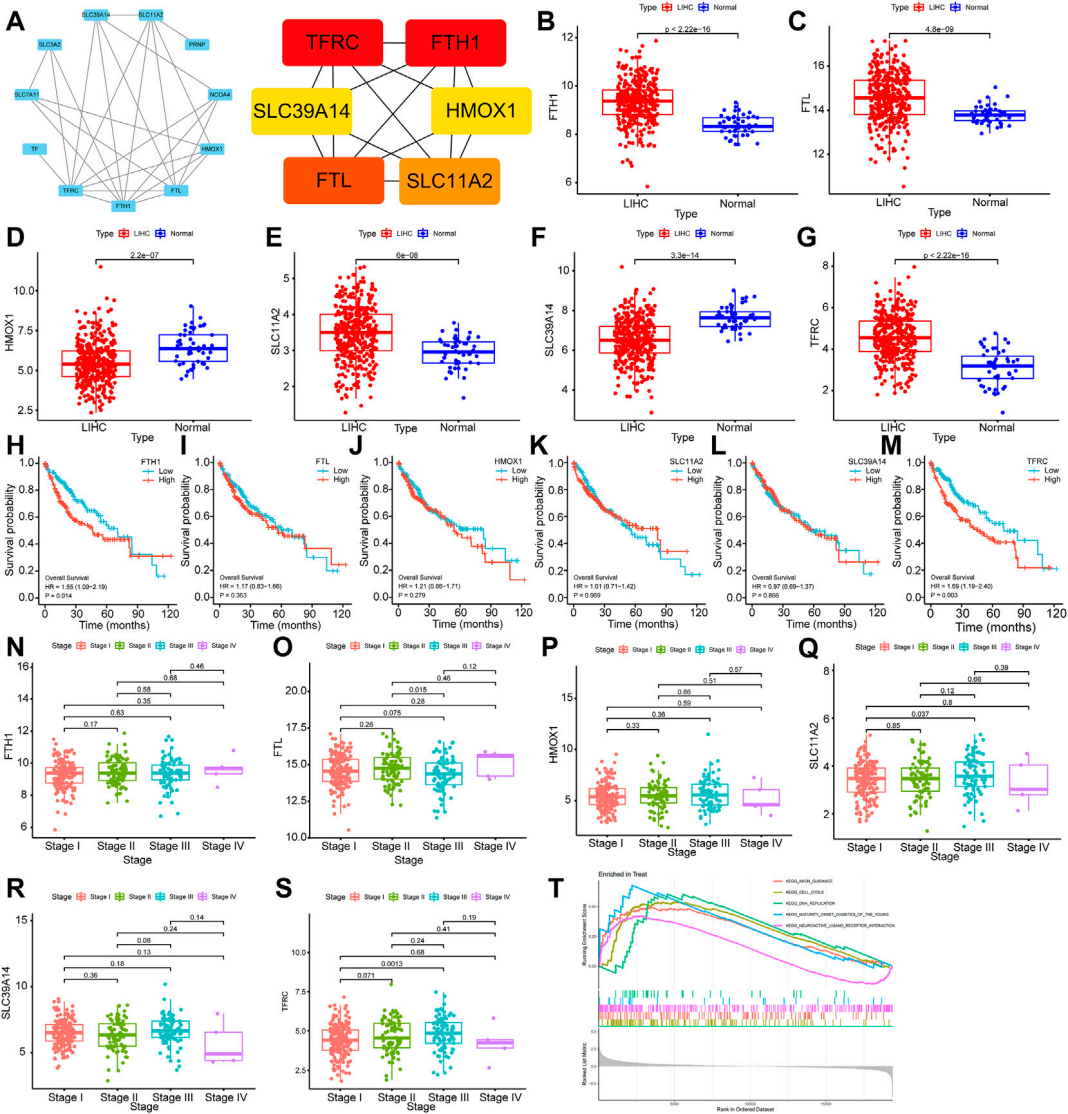
Bioinformatics analysis of ferroptosis-related genes **(A)**: Hub gene screening in the PPI network. **(B–G)**: Differential expression analysis of FTH1, FTL, HMOX1, SLC11A2, SLC39A14 and TFRC. **(H–M)**: Relationship analysis of FTH1, FTL, HMOX1, SLC11A2, SLC39A14 and TFRC and survival of HCC. **(N–S)**:Relationship analysis of FTH1, FTL, HMOX1, SLC11A2, SLC39A14 and TFRC and clinical stages of HCC. **(T)**: GSEA results of TFRC.

Differentially expressed proteins were input into the STRING database for PPI analysis, and the PPI network was plotted using Cytoscape software. Six key genes were screened with the Degree algorithm of CytoHubba, including TFRC, FTH1, FTL, SLC11A2, SLC39A14, and HMOX1 (Figure 7A).

#### 5.7.2 Differential expression analysis of hub genes in the RNA-Seq dataset of hepatic carcinoma

The transcriptional level differences of Hub genes in HCC and normal tissues were analyzed through an RNA-seq dataset. The transcriptional levels of FTH1, FTL, SLC11A2 and TFRC in the HCC group were increased compared with those in the normal group, while the transcriptional levels of HMOX1 and SLC39A14 were decreased. These differences were statistically significant (*p* < 0.01) (Figures 7B–G).

#### 5.7.3 Hub genes and clinical pathological analysis of hepatic carcinoma

To investigate the relationships of FTH1, FTL, HMOX1, SLC11A2, SLC39A14, and TFRC expression with the survival and prognosis of patients with HCC, a Kaplan‒Meier survival analysis was performed. FTH1 (*p* = 0.014, HR = 1.55, 95% Cl (1.09–2.19)) and TFRC (*p* = 0.016, HR = 1.53, 95% Cl (1.08–2.17)) were related to the survival of patients (Figures 7H–M). By analyzing the relationship between FTH1, FTL, HMOX1, SLC11A2, SLC39A14 and TFRCDACH1 and the clinical pathological stages of HCC, we found that the expression of these genes increased gradually in Stage I, Stage II and Stage III. Moreover, gene expression was statistically significant in Stage I and Stage III (*p* = 0.0013) (Figures 7N–S).

#### 5.7.4 GSEA

Gene enrichment analysis (GSEA) was performed on TFRC. According to the GSEA-KEGG results, TFRC was mainly enriched in cell cycle, DNA duplication, neural active ligand‒receptor interaction, and axon pathways leading to mature diabetes in young individuals. Specifically, the cell cycle signal transduction pathway had the highest normalized enrichment score (NES) (NES = 1.821, adjusted *p*-value<0.0002) (Figure 7T).

### 5.8 Molecular docking of dehydroabietic acid with CRLS1, ACACA and TFRC

**FIGURE 8:**
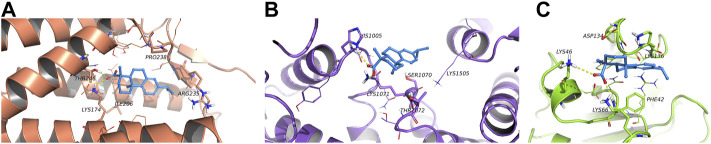
Molecular docking of dehydroabietic acid with CRLS1 **(A)**, ACACA **(B)** and TFRC **(C)**.

According to the molecular docking results (Figure 8), all three proteins could bond with dehydroabietic acid and form stable interactions. The details were as follows. In ACACA, the dehydroabietic acid-protein docking score was −7.355 kcal/mol. Carboxy groups of dehydroabietic acid formed hydrogen bonds with His1005 and Lys1071. In CRLS1, the dehydroabietic acid-protein docking score was −8.381 kcal/mol. The carboxy groups of dehydroabietic acid formed hydrogen bonds with Thr246 and Lys174. In TFRC, the dehydroabietic acid-protein docking score was −7.739 kcal/mol. The carboxy groups of dehydroabietic acid formed hydrogen bonds with Lys66 and Lys46. These data and interactions confirmed that dehydroabietic acid could bond with all 3 proteins.

### 5.9 Protein expression of ACACA and TFRC

**FIGURE 9:**
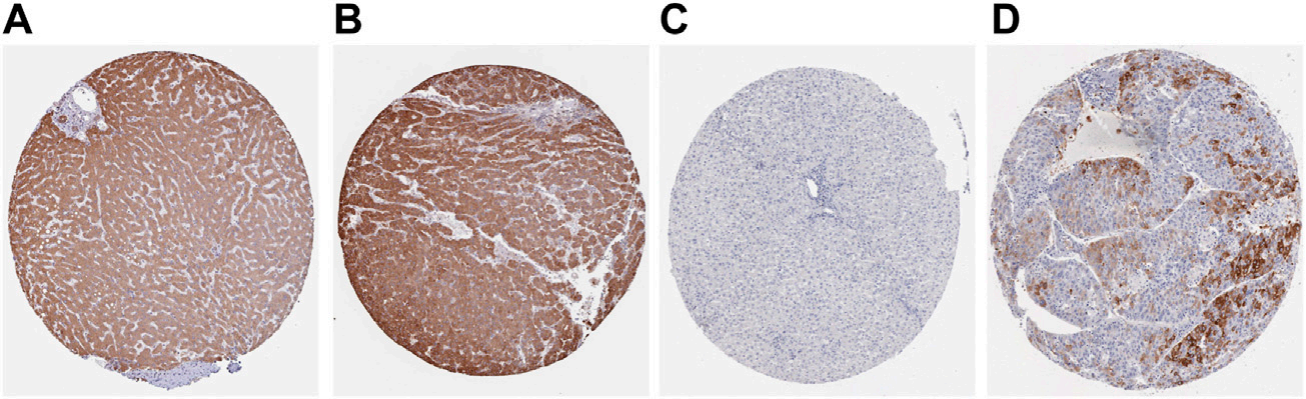
The expression levels of ACACA and TFRC were verified at the translation level based on the Human Protein Atlas database. **(A)**. ACACA protein level in normal tissues (staining: moderate; intensity: moderate; antibody No.: HPA063018). **(B)**. ACACA protein level in HCC tissues (staining: high; intensity: strong; antibody No.: HPA063018). **(C)**. TFRC protein level in normal tissues (staining: high; intensity: strong; antibody No.: CAB000153); **(D)**. TFRC protein level in HCC tissues (staining: N/A; intensity: negative; antibody No.: CAB000153).

The translation levels of ACACA and TFRC in HCC and normal tissues were analyzed through the HPA dataset. The translation levels of ACACA and TFRC in HCC tissues were higher than those in normal tissues. (Figure 9).

### 5.10 Dehydroabietic acid inhibits gene expression

**FIGURE 10:**
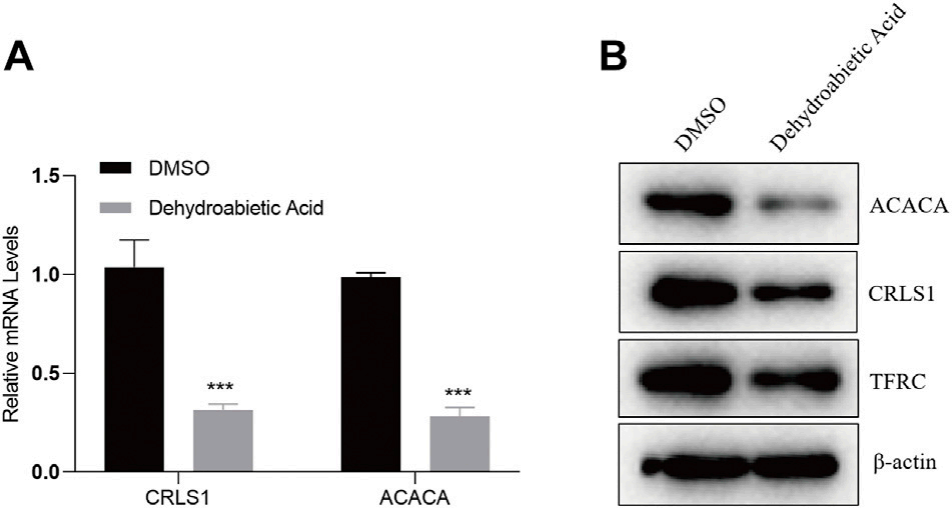
Dehydroabietic acid inhibits the expression of genes. **(A)**. qPCR detection of related gene expression after HepG2 cells were pretreated with 23.22 μg/ml dehydroabietic acid for 12 h. **(B)**. WB assay results for the related gene expression after HepG2 cells were pretreated with 23.22 μg/ml dehydroabietic acid for 12 h.

To explore the specific molecular mechanism by which dehydroabietic acid inhibits HepG2 growth, HepG2 cells were treated with 23.22 μg/ml dehydroabietic acid for 12 h. Expression levels of CRLS1, ACACA and TFRC were detected by qPCR and WB assays. According to the qPCR results, the mRNA expression levels of CRLS1 and ACACA in HepG2 cells preprocessed by dehydroabietic acid decreased (Figure 10A). According to the WB results, the protein expression levels of CRLS1, ACACA and TFRC in HepG2 cells pretreated with dehydroabietic acid decreased (Figure 10B).

## 6 Discussion

In the early period, the absolute bioavailability of dehydroabietic acid in the rat body was also studied. Dehydroabietic acid was administered orally (10 mg/kg) and *via* tail vein injection (1 mg/kg). The dehydroabietic acid concentration in plasma was tested by liquid chromatography‒mass spectrometry (LC‒MS). The pharmacokinetic parameters and absolute bioavailability using the two administration modes were calculated. After oral administration and tail intravenous injection of dehydroabietic acid, the major pharmacokinetic parameter peak concentrations (C_max_) in rats were 4993.4 ± 958.1 and 5928.0 ± 803.2 ng mL^−1^, respectively. The half-life periods (t1/2) were 1.1 ± 0.0 and 1.0 ± 0.0 h, respectively. The areas below the drug concentration in the blood-time curve (AUC_0-t_) were 7419.5 ± 976.0 and 4444.5 ± 455.3 ng h mL^−1^, respectively. The absolute bioavailability after oral administration and tail intravenous injection of dehydroabietic acid was 16.5%. Dehydroabietic acid could also inhibit the growth of hepatoma carcinoma cells. It inhibited cell growth clearly at concentrations from 3.125–800 μg/ml in a concentration-dependent manner.

In this study, proteomics identified 260 upregulated and 961 downregulated proteins, of which SLC11A2, SLC39A14 and GPX4 were significantly downregulated. The top five significantly enriched pathways were iron death, oxidative phosphorylation, and protein processing in the endoplasmic reticulum. According to published studies, the SLC11A2 polymorphism is directly related to the risk of endometrial cancer, indicating that SLC11A2 might participate in the progression of tumors (Michalczyk et al., 2022). In renal carcinoma, circ_001842 strengthens the proliferation, migration and invasion of cancer cells by inhibiting miR-502-5p and increasing the expression of SLC39A14 (Zeng et al., 2020). Glutathione peroxidase 4 (GPX4) is a major inhibitor of ferroptosis. Some studies have demonstrated that ketamine can inhibit GPX4 expression through the target lncRNA PVT1/miR-214-3p axis, thus inhibiting HCC cells (He et al., 2021). Therefore, the proteomics results showed that dehydroabietic acid could inhibit the development of liver cancer by influencing the death of cellular iron. Lipidomics screening identified 30 significantly differentially expressed metabolites; differential expression of lipids was mainly related to glycerol and phospholipid metabolism; differential metabolite level clustering results showed that it was mainly clustered into two categories and compared with the control group, the test group SQDG (26:4/18:5), PI (2:0/21:0), HBMP (16:3/16:4/16:4), and SM (SMD 14:1/27:1) metabolic capacity was significantly upregulated while PC(14:0e/16:1) metabolic level was significantly reduced. Studies have shown that SQDG is able to inhibit angiogenesis in tumor tissue and tumor cell proliferation (Maeda et al., 2008). The presence of metabolic abnormalities of PI, SM and PC in liver cancer tissues suggests that these metabolic abnormalities may be involved in the development of liver cancer (Assi et al., 2018). European metabolic data showed that high SM metabolic levels and low PC metabolic levels reflect metabolic characteristics of healthy living, which are inversely associated with the risk of liver cancer development (Li et al., 2017). Therefore, the change in the level of major metabolites in this study indicated that dehydroabietic acid could inhibit the development of hepatocellular carcinoma cells.

Cardiolipin is a type of phospholipid with specific mitochondria. It mainly exists in the mitochondrial inner membrane and is an important component in maintaining the mitochondrial structure (Chicco and Sparagna, 2007). Cardiolipin has a dimer structure, in which two groups of phosphatidic acids are connected by a central glycerol molecule (Schlame et al., 2000). Cardiolipin mainly exists in the mitochondrial inner membrane and accounts for approximately 25% of the total phospholipid content. Approximately 65% of cardiolipin on the mitochondrial inner membrane is located in the endite, and the rest is located in the exite. There is approximately 4% cardiolipin on the mitochondrial outer membrane, especially in contact positions close to the inner and outer membranes. At inner-outer membrane interfaces, cardiolipin can reach the mitochondrial outer membrane and surface of mitochondria close to the cytoplasm (Paradies et al., 2014). According to Chinese and foreign-associated studies, cardiolipin has a unique aliphatic acyl side chain structure, which is very important in the development of the biological functions of cardiolipin (Sparagna et al., 2007). When the pathological state causes oxidative stress, high molecular weights of polyunsaturated fatty acid acyl side chains, such as arachidonic acid and docosahexaenoic acid, might replace cardiolipin. These replaced side chains further cause oxidation of cardiolipin and thereby induce pathological changes. As a result, electron transport complex I in mitochondria and its activity are damaged, thus leading to mitochondrial dysfunction (Razak and Anand, 2004). Acyl coenzyme A synthesizes long chain acyl-CoA synthetases (ACSLs), which are indispensable enzymes in organisms. ACSLs have five family members: ACSL1, ACSL3, ACSL4, ACSL5 and ACSL6. Among them, ACSL3 is the member with the highest expression and complex functions (Mashek et al., 2004). ACSL3 can activate unsaturated fatty acids with a carbon chain length of 16–20 as well as −5,8,11,14-eicosatetraenoic acids and produce lecithin and lipid droplets. Lecithin exists on the surface of very-low-density lipoprotein and is an important component. Lipid droplets are major organelles for storing neural lipids in cells, and they mainly maintain the dynamic balance of lipids (Kageyama et al., 2013). Some studies have demonstrated that when ACSL3 participates in the synthesis and autophagy of lipid droplets, ACSL3 can regulate the steady state of lipids in cells (Lv et al., 2019). According to comprehensive multiomics analysis results, real-time quantitative PCR and ELISA, ACSL3 participates in cardiolipin metabolism.

According to previous studies, CRLS1 can also be used as a tumor suppressor to decrease thrombogenesis in tumors. The long-chain noncoding RNA LINC01272 inhibits the proliferation of lung cancer cells and promotes apoptosis by modulating the miR-7-5p/CRLS1 signaling axis (Ma et al., 2021). ACC is an essential rate-limiting enzyme in fatty acid metabolism and has become an attractive target of various metabolic diseases due to its critical regulatory role in fatty acid synthesis and oxidative pathways (Chen et al., 2019). In addition, ACC also participates in the progression of tumors. Some studies have pointed out that in breast cancer, TGFβ-activated kinase (TAK) catalyzes the phosphorylation of ACC and thereby activates the transcription of Smad2, ultimately influencing the metastasis of breast cancer. Mice with ACC1 deficiency easily suffer recurrent tumors after the first tumor excision (Rios et al., 2017). TFRC can combine with transferrin, which carries free iron to transfer iron out of and into cells, thus increasing the iron level in cells (Jiang et al., 2021). Upregulated TFRC expression has been found in various tumors, including HCC, indicating that TFRC-related pathways play an important role in the progression of tumors (Kindrat et al., 2016). The key protein TFRC was screened through a deep proteomics analysis of HCC cells treated with dehydroabietic acid. It found that TFRC was significantly different in different clinical stages of patients with HCC. Moreover, through molecular docking, this study demonstrated that dehydroabietic acid could bind with CRLS1, ACACA and TFRC, indicating that dehydroabietic acid could kill HCC cells effectively by regulating ferroptosis and lipidosomes, especially cardiolipin metabolism.

## 7 Conclusion

Changes in the protein and lipid levels in HepG2 cells after administration of dehydroabietic acid, especially those that changed significantly, were analyzed through proteomics and lipidomics studies. Moreover, the specific regulatory functions of proteins and lipids were analyzed. Conjoint analysis of ferroptosis-related proteins and significantly changed lipids was performed. According to experimental verification, ACSL3 knockdown facilitated the accumulation of cardiolipin in the cell supernatant and lysate.

## Data Availability

The datasets presented in this study can be found in online repositories. The names of the repository/repositories and accession number(s) can be found below: ProteomeXchange, PXD036015.
